# Genetic Variation in the *ZNF208* Gene at rs8103163 and rs7248488 Is Associated With Laryngeal Cancer in the Northwestern Chinese Han Male

**DOI:** 10.3389/fgene.2022.813823

**Published:** 2022-04-11

**Authors:** Shiyang Wang, Xiulin Wen, Ruimin Zhao, Yanxia Bai

**Affiliations:** ^1^ Department of Otolaryngology Head and Neck Surgery, The First Affiliated Hospital of Xi’an Jiaotong University, Xi’an, China; ^2^ Department of Nursing, The First Affiliated Hospital of Xi’an Jiaotong University, Xi’an, China

**Keywords:** ZNF208 gene, genetic variants, laryngeal cancer, Northwestern Chinese Han male, case-control study

## Abstract

**Background:** Laryngeal cancer is more common in middle-aged and older men. We conducted an association analysis between *ZNF208* polymorphisms and laryngeal cancer (LC) risk in the Northwestern Chinese Han male.

**Methods:** A total of 352 subjects (172 LC patients and 180 controls) were involved in this study. Agena MassARRAY was used to determine the genotypes. Unconditional logistic regression analysis was performed to explore the relevance.

**Results:** Two SNPs were associated with the risk of LC: rs8103163, OR = 1.41, *p* = 0.043; rs7248488, OR = 1.45, *p* = 0.025. Furthermore, rs8103163 was associated with an increased risk of LC under a log-additive model (OR = 1.40, *p* = 0.042), and rs7248488 was related to a higher risk of LC under a recessive model (OR = 2.33, *p* = 0.025) and a log-additive model (OR = 1.44, *p* = 0.026).

**Conclusions:** We first demonstrated that the rs8103163 A allele and the rs7248488 A allele in *ZNF208* create susceptibility to laryngeal cancer in the Northwestern Chinese Han male.

## Introduction

Laryngeal cancer (LC) is a common type of malignant tumor in the head and neck, and is the seventh most frequent cancer type worldwide (ChowM: HeadNeck Cancer, 2020). The average age of onset of the disease is 64 years, and it is more common in males than in females (van der Kamp et al., 2021; Mazul et al., 2021). The etiology and pathogenesis of LC are still undefined, but multiple research studies supported that LC is the result of the combined effects of multiple factors, primarily environmental and genetic factors. Alcohol and tobacco consumption are well-recognized risk factors for LC (Ramroth et al., 2004; An et al., 2015; Chen et al., 2016). It is reported that cancer risk is directly related to duration and number of cigarettes smoked and alcohol consumed (Abbasi et al., 2010). A few epidemiologic studies have investigated the risk of LC in subjects with family history of cancer. A study based on a US population database showed that the LC risk of the subjects with LC family history increased 8.0 fold in 246 LC cases (Goldgar et al., 1994). The International Head and Neck Cancer Epidemiology Consortium (INHANCE) data from 12 case-control studies showed that on a total of 2,357 LC cases, the risk of LC would be increasing 2.1 fold in subjects reporting a first-degree relative with a head and neck cancer (Negri et al., 2010).

The Zinc finger protein 208 (ZNF208) gene is located in the p12 region of human chromosome 19. ZNF208, a member of the Zinc finger protein, can recognize and interact with special DNA sequences *via* a sequence of zinc finger motifs and regulate gene transcription (Jin et al., 2015). There have been observed mutations of ZNF208 in gastric carcinoma (Zhang et al., 2015a). Also, this gene was in connection with the response to imatinib mesylate treatment in patients with gastrintinal stromal tumors (Group et al., 2009). Recently, rs8105767 variant within ZNF208 gene has attracted much attention in a genome-wide association study related to its relevance to telomere length (Alves-Paiva et al., 2019). This variant was reported to be related to the risk of glioma and soft tissue sarcoma in a genome-wide association study related to its relevance to telomere length (Vaiciulis et al., 2020; Gabriel et al., 2009). Nevertheless, this SNP was not associated with reduced telomere length in squamous cell carcinoma of the head and neck in a Chinese Han population (Thomas et al., 2007). It has been discovered that there is an association between telomere length and head and neck cancer development (Alves-Paiva et al., 2019). The attention has been increasingly focused on investigations of telomeres and telomere length regulating genes, due to their importance in aging and development of chronic diseases and malignant tumors. Paulius Vaiciulis et al. have found the association of relative leucocyte telomere length and gene single nucleotide polymorphisms (TERT, TRF1, and TNKS2) in LC (Vaiciulis et al., 2020). However, the correlation between the gene mutations of telomere length–associated gene ZNF208 and LC remains uncertain. Thus, relative genetic roles of this gene are worth digging out. In the preset study, we aimed to investigate following four SNPs, including rs2188972, rs2188971, rs8103163, and rs7248488 within *ZNF208* gene. Our research might provide more significant evidence for further elucidating the pathogenesis of LC.

## Methods

### Ethics Statement

The procedures and purpose of the study were informed both in writing and orally to all participants. Indispensably, the informed consent documents were signed by all subjects in this study. Then, the protocols for this research were approved by the Ethical Committee of the First Affiliated Hospital of Xi’an Jiaotong University, and they complied with the World Medical Association Declaration of Helsinki. All subsequent studies were analyzed in accordance with approved guidelines and regulations.

### Study Subjects

A case-control study involving a Chinese population of 172 LC patients and 180 unrelated controls was conducted at the First Affiliated Hospital of Xi’an Jiaotong University School of Medicine. By pathological analysis, all patients were confirmed LC. Exclusion criteria included a self-reported history of cancer and prior radiation and chemotherapy. Neither the age nor the disease stage was considered for cases, and control subjects had no chronic or severe endocrine and metabolic diseases. All the subjects were ethnic Han Chinese who were genetically unrelated. The sample size was determined using G*Power 3.1.9.2 software.

### SNP Genotyping

Based on the NCBI dbSNP database, we selected four candidate polymorphisms (rs2188972, rs2188971, rs8103163, and rs7248488) in *ZNF208*. Each SNP had a minor allele frequency (MAF) of at least 5% in the Han Chinese population from the 1000 Genome Projects (http://www.internationalgenome.org/). HaploReg v4.1 (https://pubs.broadinstitute.org/mammals/haploreg/haploreg.php) was used to predict the potential function. Blood samples (5 ml) were obtained from patients and controls were stored in EDTA-coated tubes. Genomic DNA extraction was carried out using the GoldMag DNA Purification Kit (GoldMag Co. Ltd., Xi’an City, China) according to the manufacturer’s protocol and then quantified by NanoDrop 2000 (Thermo Scientific, Waltham, MA, United States). If the purity of the DNA tested is between 1.7 and 1.9, it will meet the requirements of subsequent experiments, and it is stored at −20°C for further experiments. The primers for amplification and single base extension were designed using the Assay Design Suite V2.0 (https://agenacx.com/online-tools/) (Gabriel et al., 2009). These candidate SNPs were genotyped using the MassARRAY iPLEX (Agena Bioscience, San Diego, CA, United States) platform with the matrix-assisted laser desorption ionization-time of flight (MALDITOF), and it was conducted by two laboratory technicians double-blinded. Genotyping results were output using Agena Bioscience TYPER version 4.0 software (Thomas et al., 2007). The PCR primers are shown in Supplementary Table S1.

### Statistical Analysis

Student’s *t*-test was performed to analyze the differences in the age distribution between the cases and controls. Hardy–Weinberg equilibrium (HWE) was tested by an χ2 test for each SNP. Unconditional logistic regression analysis that was analysis adjustment with age was used to test OR and 95% CI (Huang et al., 2015). Four genetic models (co-dominant, dominant, recessive, and log-additive) were performed using SNPStats (http://bioinfo.iconcologia.net/snpstats/start.htm) software to estimate the relationship between each SNP and LC risk. Furthermore, Haploview software (version4.2) was used to construct haplotype and estimate the pairwise linkage disequilibrium, and the SHEsis software platform (http://analysis.bio-x.cn/myAnalysis.php) was applied to estimate the correlation between haplotype and LC risk (Yong and He, 2005). Microsoft Excel and SPSS 21.0 (SPSS, Chicago, IL, United States) were used for statistical analyses. Finally, the MDR analysis was carried out using MDR software (version 3.0.2) to evaluate the SNP–SNP interactions among these three candidate SNPs. All *p-*values were two-sided, and *p* < 0.05 was considered as statistically significant.

## Results

The mean age [±standard deviation (SD)] at diagnosis was 60.78 ± 10.05 years in the cases and 60.25 ± 5.48 years in the controls. The distribution of selected characteristics of the patients and age-matched controls are shown in Table 1. Among 172 LC cases with available tumor location, lymph node metastasis, and smoking or no smoking information, 55 (31.98%) cases were lymph node metastasis and 135 (78.49%) cases were smoking.

**TABLE 1:** Characteristics of the cases and controls included in the study.

Characteristics		Case (%)	Control (%)	*p*
Number		172	180	
Gender	Male	172	180	
Age (years), mean ± SD		60.78 ± 10.05	60.25 ± 5.48	0.538
Lymph node metastasis	No	117 (68.02)		
	Yes	55 (31.98)		
Smoking	No	37 (21.51)		
	Yes	135 (78.49)		

To evaluate the function of the selected SNPs (rs2188972, rs2188971, rs8103163, and rs7248488), we used HaploReg to annotate the functions of SNPs in *ZNF208*. Then the analysis results of the allele model indicated that these two variants were associated with LC (rs8103163, OR = 1.41, 95%CI: 1.00–1.94, *p* = 0.043; rs7248488, OR = 1.45, 95%CI: 1.04–2.01, *p* = 0.025); and there was no statistical significance found in the variants of rs2188972 and rs2188971 (Table 2).

**TABLE 2:** Allele frequencies of the four SNPs in *ZNF208*.

SNP_ID	Chr.	Position	Allele	HaploReg	MAF		HWE *p* value	OR (95%CI)	*p*	FDR test
					Case	Control				
rs2188972	19p12	Intron	A/G	Motifs changed, selected eQTL hits	0.515	0.485	0.654	1.22 (0.91–1.65)	0.178	0.445
rs2188971	19p12	Intron	T/C	Selected eQTL hits	0.301	0.251	1.000	1.28 (0.91–1.79)	0.146	0.243
rs8103163	19p12	Intron	A/C	Motifs changed, selected eQTL hits	0.322	0.253	1.000	**1.41 (1.00–1.94)**	**0.043**	0.108
rs7248488	19p12	Intron	A/C	Motifs changed, selected eQTL hits	0.329	0.253	1.000	**1.45 (1.04–2.01)**	**0.025**	0.063

MAF, Minor allele frequency; HWE, Hardy-Weinberg equilibrium; OR, odds ratio; 95%CI: 95% confidence interval. *p* < 0.05 indicates statistical significance. Bold values indicate a significant difference.

We evaluated the correlation between the *ZNF208* SNPs and LC susceptibility using multiple genetic models by hypothesizing that the minor allele of each polymorphism was a risk factor, and the results are shown in Table 3. We observed that *ZNF208* rs8103163 was linked to an increased risk of LC based on the results of the log-additive model (adjusted OR = 1.40, 95%CI: 1.01–1.94, *p* = 0.042), and *ZNF208* rs7248488 performed an increasing-risk effect of LC under the recessive model (adjusted OR = 2.33, 95%CI: 1.09–4.97, *p* = 0.025) and the log-additive model (adjusted OR = 1.44, 95%CI: 1.44–2.00, *p* = 0.026) adjusted by age. The rs7248488 was still performing an increasing-risk effect of LC under the log-additive model (*p* = 0.043) after FDR analysis. Furthermore, we stratified our study subjects based on smoking stratification to investigate the association of the four candidate SNPs with LC risk. However, no statistical significance was found between four loci in *ZNF208* with LC risk without or with FDR analysis, as shown in Table 4.

**TABLE 3:** Genotypic model analysis of the relationship between the *ZNF208* SNPs and the risk of LC.

Model	Genotype	Control	Case	Adjusted by age		
				OR (95%CI)	*p*	FDR test
rs2188972	—					
Codominant	G/G	50 (27.8%)	37 (21.6%)	1	0.350	0.350
	G/A	93 (51.7%)	92 (53.8%)	1.33 (0.80–2.23)		
	A/A	37 (20.6%)	42 (24.6%)	1.55 (0.84–2.87)		
Dominant	G/G	50 (27.8%)	37 (21.6%)	1	0.180	0.300
	G/A-A/A	130 (72.2%)	134 (78.4%)	1.39 (0.86–2.27)		
Recessive	G/G-G/A	143 (79.4%)	129 (75.4%)	1	0.340	0.425
	A/A	37 (20.6%)	42 (24.6%)	1.28 (0.77–2.11)		
Log-additive	---	---	---	1.25 (0.92–1.70)	0.160	0.800
rs2188971						
Codominant	C/C	100 (55.9%)	81 (50.3%)	1	0.260	0.325
	C/T	68 (38%)	63 (39.1%)	1.15 (0.73–1.80)		
	T/T	11 (6.2%)	17 (10.6%)	1.95 (0.86–4.40)		
Dominant	C/C	100 (55.9%)	81 (50.3%)	1	0.290	0.290
	C/T-T/T	79 (44.1%)	80 (49.7%)	1.26 (0.82–1.93)		
Recessive	C/C-C/T	168 (93.8%)	144 (89.4%)	1	0.130	0.650
	T/T	11 (6.2%)	17 (10.6%)	1.83 (0.83–4.05)		
Log-additive	---	---	---	1.28 (0.92–1.79)	0.140	0.350
rs8103163						
Codominant	C/C	100 (55.6%)	81 (47.4%)	1	0.097	0.121
	C/A	69 (38.3%)	70 (40.9%)	1.25 (0.81–1.96)		
	A/A	11 (6.1%)	20 (11.7%)	2.30 (1.04–5.10)		
Dominant	C/C	100 (55.6%)	81 (47.4%)	1	0.120	0.120
	C/A-A/A	80 (44.4%)	90 (52.6%)	1.40 (0.92–2.13)		
Recessive	C/C-C/A	169 (93.9%)	151 (88.3%)	1	0.056	0.093
	A/A	11 (6.1%)	20 (11.7%)	2.09 (0.97–4.51)		
Log-additive	---	---	---	**1.40 (1.01–1.94)**	**0.042**	0.210
rs7248488						
Codominant	C/C	100 (55.6%)	80 (47.1%)	1	0.052	0.065
	C/A	69 (38.3%)	68 (40%)	1.24 (0.79–1.94)		
	A/A	11 (6.1%)	22 (12.9%)	2.56 (1.17–5.60)		
Dominant	C/C	100 (55.6%)	80 (47.1%)	1	0.100	0.100
	C/A-A/A	80 (44.4%)	90 (52.9%)	1.42 (0.93–2.16)		
Recessive	C/C-C/A	169 (93.9%)	148 (87.1%)	1	**0.025**	0.063
	A/A	11 (6.1%)	22 (12.9%)	**2.33 (1.09–4.97)**		
Log-additive	---	---	---	**1.44 (1.04–2.00)**	**0.026**	**0.043**

OR, odds ratio; CI, confidence interval. *p* values were calculated by Wald test by unconditional logistic regression adjusted by age. Bold values indicate a significant difference (*p* < 0.05).

**TABLE 4:** Association analysis result between the ZNF208 SNPs and the risk of LC based on the smoking stratification.

Model	Genotype	Smoking = no	Smoking = yes	OR (95%CI)	*p*-value	FDR test
rs2188972						
Codominant	G/G	24 (26.1%)	45 (23.7%)	1	0.310	0.413
	G/A	53 (57.6%)	99 (52.1%)	1.00 (0.55–1.81)		
	A/A	15 (16.3%)	46 (24.2%)	1.64 (0.76–3.51)		
Dominant	G/G	24 (26.1%)	45 (23.7%)	1	0.660	0.660
	G/A-A/A	68 (73.9%)	145 (76.3%)	1.14 (0.64–2.02)		
Recessive	G/G-G/A	77 (83.7%)	144 (75.8%)	1	0.120	0.480
	A/A	15 (16.3%)	46 (24.2%)	1.64 (0.86–3.13)		
Log-additive	---	---	---	1.25 (0.87–1.81)	0.230	0.460
rs2188971						
Codominant	C/C	49 (56.3%)	96 (51.9%)	1	0.150	0.300
	T/C	35 (40.2%)	71 (38.4%)	1.04 (0.61–1.76)		
	T/T	3 (3.5%)	18 (9.7%)	3.06 (0.86–10.90)		
Dominant	C/C	49 (56.3%)	96 (51.9%)	1	0.490	0.490
	T/C-T/T	38 (43.7%)	89 (48.1%)	1.20 (0.72–2.00)		
Recessive	C/C-T/C	84 (96.5%)	167 (90.3%)	1	0.053	0.212
	T/T	3 (3.5%)	18 (9.7%)	3.02 (0.86–10.53)		
Log-additive	---	---	---	1.32 (0.87–1.99)	0.190	0.253
rs8103163						
Codominant	C/C	49 (53.9%)	96 (50.3%)	1	0.420	0.560
	C/A	37 (40.7%)	76 (39.8%)	1.05 (0.62–1.77)		
	A/A	5 (5.5%)	19 (9.9%)	1.94 (0.68–5.51)		
Dominant	C/C	49 (53.9%)	96 (50.3%)	1	0.570	0.570
	C/A-A/A	42 (46.1%)	95 (49.7%)	1.15 (0.70–1.90)		
Recessive	C/C-C/A	86 (94.5%)	172 (90%)	1	0.190	0.760
	A/A	5 (5.5%)	19 (9.9%)	1.90 (0.69–5.26)		
Log-additive	---	---	---	1.22 (0.82–1.81)	0.320	0.640
rs7248488						
Codominant	C/C	49 (54.4%)	95 (49.7%)	1	0.300	0.400
	C/A	36 (40%)	75 (39.3%)	1.07 (0.63–1.82)		
	A/A	5 (5.6%)	21 (11%)	2.17 (0.77–6.10)		
Dominant	C/C	49 (54.4%)	95 (49.7%)	1	0.460	0.460
	C/A-A/A	41 (45.6%)	96 (50.3%)	1.21 (0.73–2.00)		
Recessive	C/C-C/A	85 (94.4%)	170 (89%)	1	0.130	0.520
	A/A	5 (5.6%)	21 (11%)	2.10 (0.77–5.76)		
Log-additive	---	---	---	1.27 (0.86–1.89)	0.220	0.440

**FIGURE 1:**
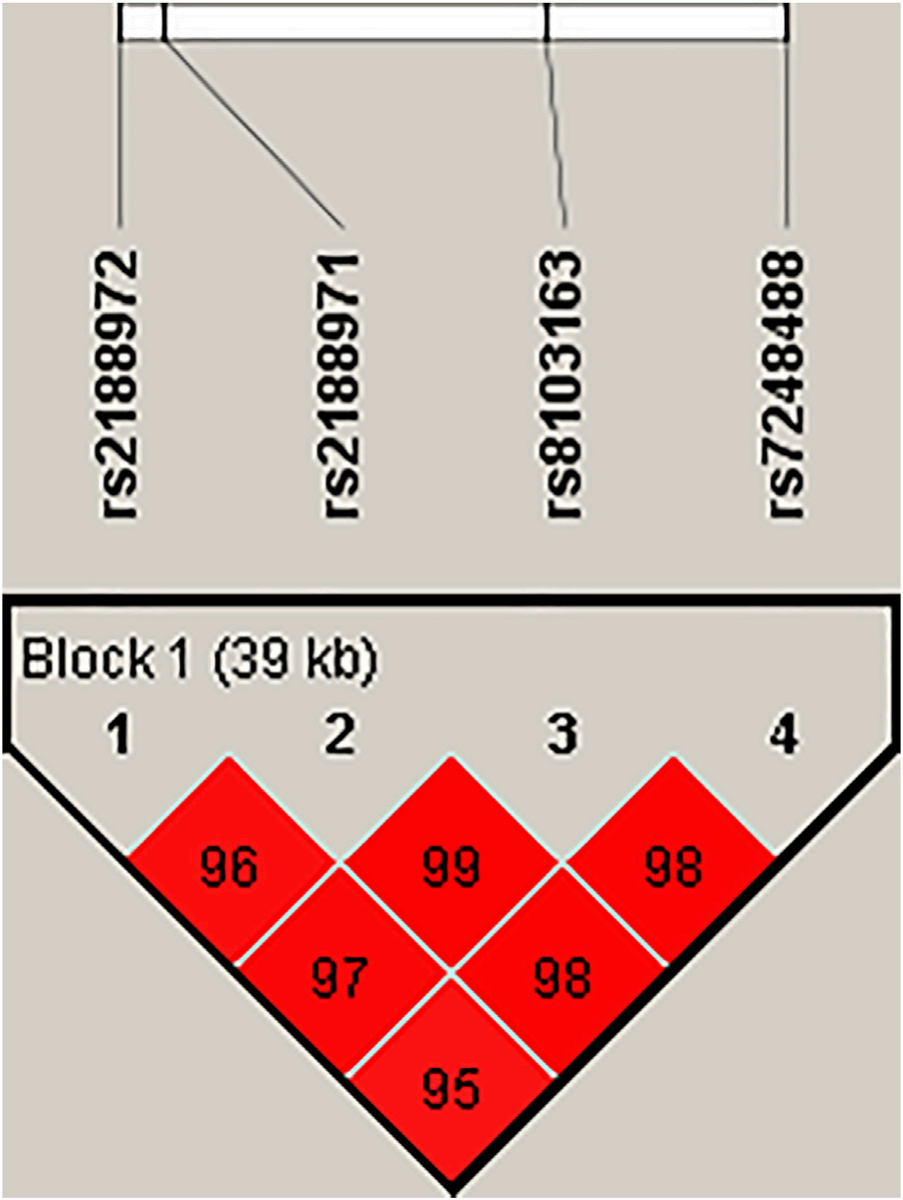
Haplotype block map for SNPs of the *ZNF208* gene. Linkage disequilibrium plots containing 4 SNPs from *ZNF208*. Red squares display statistically significant associations between a pair of SNPs, as measured by D’; darker shades of red indicate higher D’.

The LD and corresponding haplotypes’ analysis were further investigated using Haploview software. Four SNP polymorphisms (rs2188972, rs2188971, rs8103163, and rs7248488) were mapped to a 39-kb LD block and showed three haplotypes with frequencies of more than 0.05 in our subjects. In Figure 1, the red squares of the *ZNF208* LD block presented significant linkage between the four SNPs. Regrettably, none of the haplotypes was related to the incidence of LC (*p* > 0.05, Supplementary Table S2).

**FIGURE 2:**
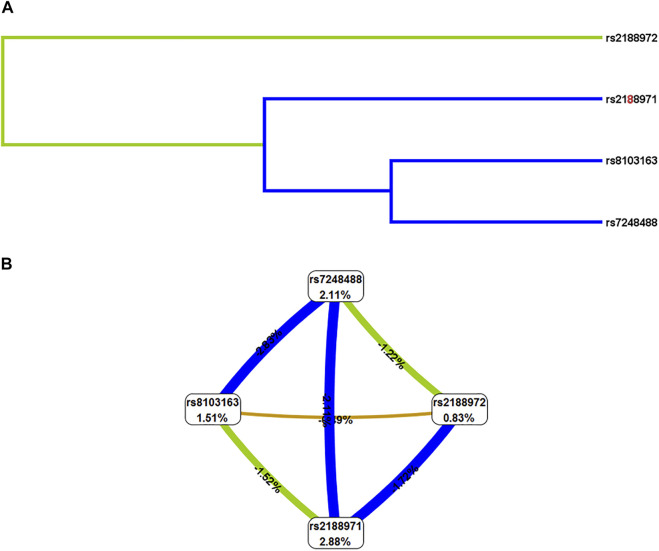
SNP-SNP interaction of four SNPs on *ZNF208* gene. **(A)** Dendrogram of SNP-SNP Interaction. The shorter the line connecting the 2 SNPs, the stronger the interaction. **(B)** Fruchterman-Reingold of interactions between the four genetic variants. Green and blue represent redundancy or correlation. Contrarily, the redder the color, the more the synergy effect between those four SNPs.

Finally, we conducted the MDR analysis to explore the SNP–SNP interaction among the four loci (rs2188972, rs2188971, rs8103163, and rs7248488) in the *ZNF208* to better evaluate the effect of *ZNF208* variants on LC risk (Table 5). A model consisting of four loci (rs2188972, rs2188971, rs8103163, and rs7248488) with the largest “CV consistency” value (10/10) could be the best multilocus model. At the same time, the impact of this best model on the risk of LC prediction was significant (rs2188972-AG, rs2188971-TC, rs3931698-GT, rs8103163-AC, and rs7248488-AC). At the same time, the impact of this best model on the risk of LC prediction was significant (*p* < 0.01). Likewise, as shown in Figure 2, the bluer the string color, the greater the redundancy effect among those four SNPs. Contrarily, the redder the color, the greater the synergy effect among those four SNPs. Furthermore, we could observe that a strong redundancy effect existed between rs2188971, rs8103163, and rs7248488.

**TABLE 5:** MDR analysis for impact of *ZNF208* variants on risk of LC.

Model	Training bal. acc.	Testing bal. acc.	CV consistency	Accuracy	Sensitivity	Specificity	OR (95%CI)	*p*
rs2188971	0.555	0.506	6/10	0.552	0.163	0.942	3.15 (1.48–6.71)	**0.002**
rs2188972, rs2188971	0.583	0.555	9/10	0.581	0.488	0.674	1.98 (1.28–3.06)	**0.002**
rs2188972, rs2188971, rs8103163	0.591	0.567	9/10	0.590	0.506	0.674	2.12 (1.37–3.28)	**0.001**
rs2188972, rs2188971, rs8103163, rs7248488	0.594	0.573	10/10	0.593	0.512	0.674	2.17 (1.40–3.36)	**0.001**

MDR, multifactor dimensionality reduction; bal. acc., balanced accuracy; CV, cross-validation; OR, odds ratio; CI, confidence interval. All *p* values in this study were two-tailed. Bold values indicate a significant difference (*p* < 0.05).

## Discussion

This study aimed to explore the correlations of *ZNF208* gene polymorphisms with LC susceptibility. To the best of our knowledge, this is the first study to explore the association between ZNF208 SNPs (rs2188972, rs2188971, rs8103163, and rs7248488) and LC risk. Our findings suggested that polymorphisms of rs8103163 and rs7248488 in *ZNF208* gene might be significantly correlated with LC susceptibility.

Zinc finger protein (ZNF) is the largest transcriptional regulator family, and *ZNF208* as one of these proteins plays an important role in tumorigenesis (Cheng et al., 2010). As a matter of fact, accumulating evidence has shown that ZNF208 polymorphisms are associated with several diseases. Yu et al. noted that variations in ZNF208 were associated with risk of ischemic stroke after age and sex adjustment in a recessive model (Yu et al., 2017). The rs8105767 in *ZNF208* has been shown to be relevant with the risk of neuroblastoma and lung adenocarcinoma (Zhang et al., 2015b; Walsh et al., 2016). All these studies showed that *ZNF208* gene polymorphism is related to the development of diseases. The reason was thought to be the variation of *ZNF208*’s close relationship with telomeres and telomerase (Walsh et al., 2016; Zhang et al., 2015b). Telomerase is a widely used tumor marker and plays an important role in the development of tumor (). However, the rs8105767 was not associated with reduced telomere length in squamous cell carcinoma of the head and neck in a Chinese population (Thomas et al., 2007). Moreover, Li et al. found that SNPs (rs2188971, rs8103163, and rs7248488) of *ZNF208* may be associated with an increased risk of hepatitis B virus (HBV) in the Han population (). Wang et al. found that SNPs (rs8103163 and rs7248488) were associated with an increased risk of esophageal cancer (Li et al., 2017). Our results showed a consistent direction of risk; SNPs of rs8103163 and rs7248488 in *ZNF208* increased risk of LC in the Northwestern Chinese Han male (Wang et al., 2016).

Especially, the research on *ZNF208* gene has enriched our understanding of cancer development and prompted a series of explorations in tumor research. Hirbe et al. () found that the mutation of *ZNF208* gene occurred during the transformation of neurofibroma from benign to malignant through (Hirbe et al., 2015) whole-exome sequencing. Zhang et al. found that *ZNF208* activity and mRNA expression decreased due to somatic mutations in patients with gastric cancer, suggesting that *ZNF208* plays a central role in tumor suppression (Zhang et al., 2015a). There were some correlations between the expression of *ZNF208* gene and the patients’ response to imatinib mesylate in another study of gastrointestinal stromal tumors (). Cui et al. (Cui et al., 2017) found that *ZNF208* affects the prognosis of glioma. Furthermore, our results demonstrated that SNPs (Rink et al., 2009) (rs8103163 and rs7248488) in *ZNF208* are significantly relevant to LC susceptibility (*p* = 0.043 and *p* = 0.025, respectively). Nevertheless, the detailed roles of ZNF208 or SNPs (rs2188972, rs2188971, rs8103163, and rs7248488) in LC risk remain to be explored in further study, so biological function studies are needed to validate our findings.

It is the first time that the genetic polymorphisms of *ZNF208* have been associated with LC risk based on the Northwestern Chinese Han male according to our study. Mutations at rs8103163 and rs7248488 of ZNF208 are associated with increased LC risk. These two SNPs are either intronic or 3′ UTR variants, and functionally, they do not alter the coding sequence of the protein. Nevertheless, the transcription and gene expression might be regulated and affected by these SNPs. Several studies have proved that the expression and function of mRNA were affected by SNPs (), so these SNPs might have potential function in ZNF208 expression level. Through the GTEx Portal database, we found that rs8103163 and rs7248488 were significantly expressed in a variety of tissues. Therefore, we suspected that these two sites might affect the function of ZNF208 gene, but further studies are needed.

This study still has limitations. Presently, the sample size is small. A large number of samples were needed to provide strong evidence for the results and compare with different populations. Second, the relationship between variables such as clinical indicators and LC risk was not performed because the relevant data are incomplete. Third, LC is a heterogeneous disease with many other risk factors, and we did not investigate these interactions because of the limited data. Last but not least, more functional and molecular mechanism research is warranted in the future. Despite the limitations mentioned above, our present results provided scientific evidence of ZNF208 genes with LC in the future studies.

## Conclusion

In summary, our findings showed a relationship between *ZNF208* polymorphisms and the increased risk of LC in the Chinese Northwest Han male population, which might provide potential theoretical basis for the study of LC.

This study was approved by the ethics committee of the First Affiliated Hospital of Xi’an Jiaotong University. All procedures performed in studies involving human participants were in accordance with the ethical standards of the institutional and/or national research committee and with the 1964 Helsinki Declaration and its later amendments or comparable ethical standards. All participants were informed both in writing and verbally about the procedures and purpose of the study and signed informed consent documents.

## Data Availability

The original contributions presented in the study are included in the article/Supplementary Material; The data presented in the study are deposited in the Zenodo repository, accession number (https://doi.org/10.5281/zenodo.6378127).
