# Supplementary material for: Genetic Variation in the ZNF208 Gene at rs8103163 and rs7248488 Is Associated With Laryngeal Cancer in the Northwestern Chinese Han Male
Source: Front Genet. 2022 Apr 11;13:813823. doi: 10.3389/fgene.2022.813823 (PMC9035488; doi:10.3389/fgene.2022.813823)
Supplement: Supplementary file 1 [file DataSheet1.docx]

**Supplementary Table 1** The PCR primers for the SNPs in *ZNF208*

| SNP_ID | 1st_PCRP | 2nd_PCRP | UEP_SEQ |
| --- | --- | --- | --- |
| rs2188972 | ACGTTGGATGGGCTTGATTGGTCAAATGGC | ACGTTGGATGATTCAGAACCTGTGCAAAGC | GACTTCTCAAAGAACTAGAAA |
| rs2188971 | ACGTTGGATGCTCTTCAAAGATCTACTTC | ACGTTGGATGCACTAAATCAGACTGCTGAG | TCCAAAACTAAAGTTGGCAAAA |
| rs8103163 | ACGTTGGATGCCAGAAGATCTGAGATAAAG | ACGTTGGATGTTTTGGGCCAAAAACTTTG | cctGCCAAAAACTTTGGCATACT |
| rs7248488 | ACGTTGGATGGTTCTCCAGGAACACTTATG | ACGTTGGATGGCAGAGTGTTTTCCTGGTTG | GTCATGATGAGAAGGGT |

**Supplementary Table 2** Haplotype analysis of four SNPs in *ZNF208* with LC risk (n=352, adjusted by Age)

| Haplotype | Frequency | OR (95%CI) | *p* |
| --- | --- | --- | --- |
| rs2188972\|rs2188971\|rs8103163\|rs7248488 |  |  |  |
| GTAA | 0.504 | 1.00 | 0.148 |
| ATAA | 0.277 | 1.37 (0.96-1.96) | 0.079 |
| ACCC | 0.201 | 1.05 (0.69-1.59) | 0.820 |

*p* < 0.05 indicates significant difference.
